# Iron accumulation typifies renal cell carcinoma tumorigenesis but abates with pathological progression, sarcomatoid dedifferentiation, and metastasis

**DOI:** 10.3389/fonc.2022.923043

**Published:** 2022-08-05

**Authors:** Christopher J. Greene, Kristopher Attwood, Nitika J. Sharma, Benjamin Balderman, Rongia Deng, Jason B. Muhitch, Gary J. Smith, Kenneth W. Gross, Bo Xu, Eric C. Kauffman

**Affiliations:** ^1^ Department of Urology, Roswell Park Comprehensive Cancer Center, Buffalo, NY, United States; ^2^ Department of Biological Sciences, University at Buffalo, Buffalo, NY, United States; ^3^ Department of Biostatistics and Bioinformatics, Roswell Park Comprehensive Cancer Center, Buffalo, NY, United States; ^4^ Department of Immunology, Roswell Park Comprehensive Cancer Center, Buffalo, NY, United States; ^5^ Department of Molecular and Cellular Biology, Roswell Park Comprehensive Cancer Center, Buffalo, NY, United States; ^6^ Department of Pathology, Roswell Park Comprehensive Cancer Center, Buffalo, NY, United States; ^7^ Department of Cancer Genetics, Roswell Park Comprehensive Cancer Center, Buffalo, NY, United States

**Keywords:** iron, Prussian Blue, renal cell carcinoma, clear cell, metastasis, ferroptosis

## Abstract

Iron is a potent catalyst of oxidative stress and cellular proliferation implicated in renal cell carcinoma (RCC) tumorigenesis, yet it also drives ferroptosis that suppresses cancer progression and represents a novel therapeutic target for advanced RCC. The von Hippel Lindau (VHL)/hypoxia-inducible factor-α (HIF-α) axis is a major regulator of cellular iron, and its inactivation underlying most clear cell (cc) RCC tumors introduces both iron dependency and ferroptosis susceptibility. Despite the central role for iron in VHL/HIF-α signaling and ferroptosis, RCC iron levels and their dynamics during RCC initiation/progression are poorly defined. Here, we conducted a large-scale investigation into the incidence and prognostic significance of total tissue iron in ccRCC and non-ccRCC patient primary tumor cancer cells, tumor microenvironment (TME), metastases and non-neoplastic kidneys. Prussian Blue staining was performed to detect non-heme iron accumulation in over 1600 needle-core sections across multiple tissue microarrays. We found that RCC had significantly higher iron staining scores compared with other solid cancers and, on average, >40 times higher than adjacent renal epithelium. RCC cell iron levels correlated positively with TME iron levels and inversely with RCC levels of the main iron uptake protein, transferrin receptor 1 (TfR1/TFRC/CD71). Intriguingly, RCC iron levels, including in the TME, decreased significantly with pathologic (size/stage/grade) progression, sarcomatoid dedifferentiation, and metastasis, particularly among patients with ccRCC, despite increasing TfR1 levels, consistent with an increasingly iron-deficient tumor state. Opposite to tumor iron changes, adjacent renal epithelial iron increased significantly with RCC/ccRCC progression, sarcomatoid dedifferentiation, and metastasis. Lower tumor iron and higher renal epithelial iron each predicted significantly shorter ccRCC patient metastasis-free survival. In conclusion, iron accumulation typifies RCC tumors but declines toward a relative iron-deficient tumor state during progression to metastasis, despite precisely opposite dynamics in adjacent renal epithelium. These findings raise questions regarding the historically presumed selective advantage for high iron during all phases of cancer evolution, suggesting instead distinct tissue-specific roles during RCC carcinogenesis and early tumorigenesis versus later progression. Future study is warranted to determine how the relative iron deficiency of advanced RCC contributes to ferroptosis resistance and/or introduces a heightened susceptibility to iron deprivation that might be therapeutically exploitable.

## Summary

Iron accumulates in RCC and its microenvironment with tumorigenesis but declines with progression, despite opposite changes in non-neoplastic renal epithelium. These results support distinct, tissue-specific roles for iron during early RCC tumorigenesis versus progression, with important therapeutic implications related to ferroptosis resistance targeting and iron deprivation susceptibility.

## Introduction

Over 75,000 new kidney cancer diagnoses are made in the US annually, with more than 90% of cases being renal cell carcinoma (RCC) (1, 2). RCC is considered the most lethal genitourinary malignancy due to a high metastasis-to-diagnosis ratio (1, 3) and is itself composed of different histologic subtypes, each with distinct genomic and mutational landscapes (4). The clear cell RCC subtype (ccRCC, 75%) accounts for the majority of RCC diagnoses and deaths, whereas common non-ccRCC subtypes include papillary RCC (pRCC, 15%) and chromophobe RCC (chRCC, 5%) (5, 6). Well-established diagnostic risk factors for RCC include male gender, tobacco use, hypertension, diabetes, obesity, and chronic kidney disease (7, 8). Although surgical extirpation cures most patients with clinically localized RCC, metastatic relapse remains a frequent challenge (9, 10). The current standard of care for patients with metastatic RCC includes mono or dual systemic therapy targeting angiogenesis and/or immune checkpoint control (9). Although these regimens significantly extend survival for patients with advanced RCC (11–13), durable drug responses are limited, and standardized clinical biomarkers to guide patient management are lacking (9, 14). Hence, there is an urgent need to better understand the dysregulated molecular biology driving RCC tumorigenesis and progression.

Iron is the most abundant transition metal in the human body and plays a central role in a multitude of critical physiologic processes including DNA replication, mitochondrial metabolism, and oxygen storage and transport (15–19). Iron’s unique chemical reactivity facilitates efficient transition between bivalent (ferrous/reduced) and trivalent (ferric/oxidized) states to produce highly reactive oxygen species (ROS) (20, 21). ROS generated specifically by iron, particularly the potent hydroxyl radical, can damage cell protein, lipid, and nucleic acid, including mutagenic DNA breaks and base modifications (22–24). Because of its potent reactivity, cellular iron is tightly regulated by a well-defined set of proteins, including the primary iron uptake receptor, transferrin receptor 1 (TfR1/TFRC/CD71), which mediates endocytosis of the serum iron carrier protein, transferrin (25–27). Intriguingly, we and others have shown that renal epithelium has the highest or among the highest body tissue levels of TfR1 and other master regulatory proteins that increase cellular free iron, underscoring the kidney’s unique role in regulating iron and oxygen levels while, perhaps, also predisposing this organ to high iron uptake (28–30). The tendency of renal epithelium for iron uptake is evidenced by demonstration that various conditions associated with RCC (hypertension, obesity, diabetes, chronic kidney disease, and hypoxia) each trigger body iron to mobilize and accumulate specifically in the kidney (31–38).

A direct role for iron in carcinogenesis is suggested by a variety of clinical and epidemiologic observations as well as preclinical animal modeling experiments (39–47) and is commonly attributed to the ability of iron to induce oxidative stress–mediated genetic and epigenetic alterations (22–24, 39). Furthermore, iron’s role as an essential co-factor for the rate-limiting step of DNA synthesis and cell cycle progression is believed to be important for tumorigenesis (48, 49), whereas additional roles in chromatin remodeling, mitochondrial metabolism, and DNA repair may also contribute (50–53). Accordingly, conventional understanding has long been that high iron is selectively advantageous to cancers (39, 54). However, recent discoveries challenge the simplicity of this paradigm and suggest that increased iron levels may also suppress cancer through a regulated cell death process known as ferroptosis (55–58). Ferroptosis, which is morphologically and molecularly distinct from apoptosis, utilizes iron-dependent oxidative stress to induce cell suicide in response to ROS levels that overwhelm cellular antioxidant defenses (55). Oxidative stress that suppresses transformed cell properties may become a liability for tumor progression and metastasis (59–63), and resistance to iron-dependent oxidative stress may therefore be necessary for cancer progression (39, 56–58). However, the implication that tumor iron levels may thus have opposite roles during initial carcinogenesis and early tumorigenesis *vs*. later progression remains to be thoroughly explored.

The current study investigates tissue iron levels in patients with RCC based on mounting evidence of a unique role for iron accumulation in this cancer type. RCC diagnosis is significantly increased in iron/steel occupations (45–47), certain medical conditions associated with renal iron accumulation (64–66), and individuals with a polymorphism in the *TFRC* iron importer gene (67). In rodents, systemic administration of high levels of oxidized iron induces renal epithelial iron deposition and oxidative stress injury (68) that is followed months later by renal tumorigenesis with RCC histology, male predominance, and occasional lung metastasis, as similarly observed in patients with RCC (39–42). The unique cancer genetics that underlie ccRCC patient tumors further support the importance of intracellular iron concentration in this cancer. Specifically, the von Hippel Lindau (VHL)/hypoxia-inducible factor-α (HIF-α) axis, whose genetic or epigenetic inactivation underlies the vast majority of ccRCC tumors (69, 70), serves as a master axis for sensing and responding to intracellular iron levels (71–73). We recently described that VHL inactivation introduces a novel iron dependency in ccRCC cells to escape apoptosis and cell cycle arrest and that ccRCC cell lines maintain significantly higher reactive iron levels than benign renal cell lines (74). Furthermore, others have recently shown that VHL inactivation also introduces heightened susceptibility to ferroptosis; and, perhaps as a result, RCC cell lines are more sensitive to ferroptosis targeting than cell lines of other solid cancers (75, 76). Accordingly, ferroptosis resistance was highlighted at the 2020 Kidney Cancer Research Summit as among the most promising novel targets for metastatic RCC (9).

Despite the central role of iron in VHL/HIF-α signaling and ferroptosis, the fundamental question of tissue iron levels in RCC patient tissues during tumorigenesis and progression to metastasis remains unanswered. Here, we describe the first large-scale investigation to our knowledge into tissue iron levels and their prognostic significance in ccRCC and non-ccRCC patient tumor cells, the tumor microenvironment (TME), metastases, and non-neoplastic kidney tissues. Utilizing clinically annotated tissue microarrays (TMAs) from patients with RCC and a variety of other cancers, we measured tissue iron deposition and its association with RCC clinical features, including tumor pathology, TfR1 iron-importer expression, metastasis, and survival outcomes. Our findings indicate that RCC accumulates higher iron in the cancer cell compartment and TME relative to other solid cancer types or non-neoplastic kidney, but that this iron accumulation intriguingly declines with RCC pathological progression to metastasis. This work challenges the historical presumption of a solely advantageous role for iron in all phases of cancer evolution and suggests potentially different contributions to early RCC tumorigenesis versus later progression, which has important implications for the development of novel targeted therapeutic strategies in patients with RCC.

## Materials and methods

### RCC patient TMAs

Institutional review board approval at Roswell Park Comprehensive Cancer Center (RPCCC) was obtained for this study. Three multi-block TMA sets were constructed from 570 paraffin-embedded, formalin-fixed tissue specimens (primary renal cell tumors, matched non-neoplastic kidneys and/or metastases) of 286 patients who underwent radical or partial nephrectomy (N = 266) and/or metastatectomy (N = 73) for RCC or benign renal oncocytoma between 1995 and 2008 at RPCCC. Triplicate needle cores of 1.0 mm diameter were procured from representative areas of each tissue specimen and embedded in three paraffin blocks, generating nine total paraffin blocks across the three TMA sets. A 4-µm-thick section was cut from each block for Prussian Blue staining. Deidentified clinicopathologic and survival data were obtained from a prospectively maintained RPCCC nephrectomy patient database and the RPCCC cancer patient registry. RCC histologic subtype was assigned per criteria of the World Health Organization and analyzed as either ccRCC or non-ccRCC, the latter of which included pRCC, chRCC, unclassified RCC, and rarer RCC subtypes.

### Multi-cancer TMAs

A multi-cancer TMA composed of tumor tissues from various organ sites was constructed from an additional set of RPCCC patients. Triplicate needle cores of 1.0 mm diameter were procured from representative areas of each tissue specimen and embedded in three paraffin blocks. A 4-µm-thick section was generated from each block for Prussian Blue staining. Staining was analyzed for all malignant solid tumors of organs with at least five patients represented (14 total organ sites from 121 total patients). In addition to this RPCCC multi-cancer TMA, a second multi-cancer TMA was obtained from US Biomax, Inc. (Derwood, MD). This TMA harbored 1.5-mm cores from 10 different solid cancers with eight patients represented per tumor type (80 total patients). A single 5-µm-thick section from each TMA was used for Prussian Blue staining.

### Tissue iron detection

Tissue iron detection was performed using the well-described clinical assay of Prussian Blue staining (77). Staining was performed on a Dako Omnis autostainer (Agilent Technologies, Santa Clara, CA). In brief, TMA slides were deparaffinized with Clearify and rehydrated using graded alcohols. Whole section slides of liver tissue from a patient with hemochromatosis (hereditary liver iron overload) were included as a positive control for tissue iron accumulation. Target retrieval was performed using Flex TRS High pH (Agilent Technologies) for 30 min. Slides were incubated with Prussian Blue stain for 30 min and counterstained with hematoxylin for 8 min. Scoring of Prussian Blue staining levels was managed by a clinical genitourinary pathologist (BX) based on percentage tissue positivity (0%–100%) and staining intensity (0+, absent; 1+, low; 2+, moderate; 3+, high). The staining level for each tissue core was summarized by an H-score, which is the product of the percentage tissue positivity and the intensity score, as previously described (28). H-scores from replicate cores were averaged to generate the H-score of each tissue specimen. TfR1 protein immunostaining and scoring from this same TMA set was previously performed and separately reported (28).

### Statistics

Patient characteristics were summarized as frequencies and relative frequencies, with continuous variables categorized based on either clinically relevant cutoffs or dichotomization at the median. Prussian Blue staining incidence was summarized using frequencies and relative frequencies and compared between tissue types or tumor subtypes using Fisher’s exact test. Prussian Blue H-scores were summarized by tissue type (primary tumor, non-neoplastic kidney, and metastasis) using the mean and standard error (SE) and compared between tissue types in a pairwise fashion using the Mann-Whitney U-test. H-scores were compared between matched primary tumor and metastatic tissue using the Sign test. A Spearman correlation coefficient was used to compare Prussian Blue H-scores: 1) between matched primary tumors and metastasis tissues from the same patient, 2) between tumor cells and the TME within the same tissue specimen, 3) between non-neoplastic renal epithelial cells and adjacent renal stroma within the same tissue specimen, and 4) with previously reported TfR1 immunostain H-scores from the same tissue specimens (28).

Associations between Prussian Blue stain H-scores and clinicopathologic variables were evaluated using either the Mann-Whitney U or Kruskal–Wallis exact test, as appropriate. For survival analyses, Prussian blue H-scores were dichotomized at the median in the ccRCC and non-ccRCC subsets and summarized as low (at or below the median) or high (above the median). Univariate associations between low *vs*. high iron H-scores and metastasis-free, cancer-specific and overall survival were evaluated using standard Kaplan–Meier methodology, with comparisons made using a log-rank test. Cox regression models were used to obtain hazard ratios (HRs). For survival outcomes that were significantly associated with iron H-score on univariate analysis, multivariable analyses were conducted using Cox regression models that also included age (overall survival only), tumor stage, tumor grade, and tumor size. All models were fit using Firth’s method, and HRs with corresponding 95% confidence intervals (CI) were obtained from model estimates. Model assumptions were verified graphically using residual plots. All statistical analyses were conducted using SAS v9.4 (Cary, NC) and at a significance level of 0.05.

## Results

### Tissue iron levels in different cancers

**Figure 1:**
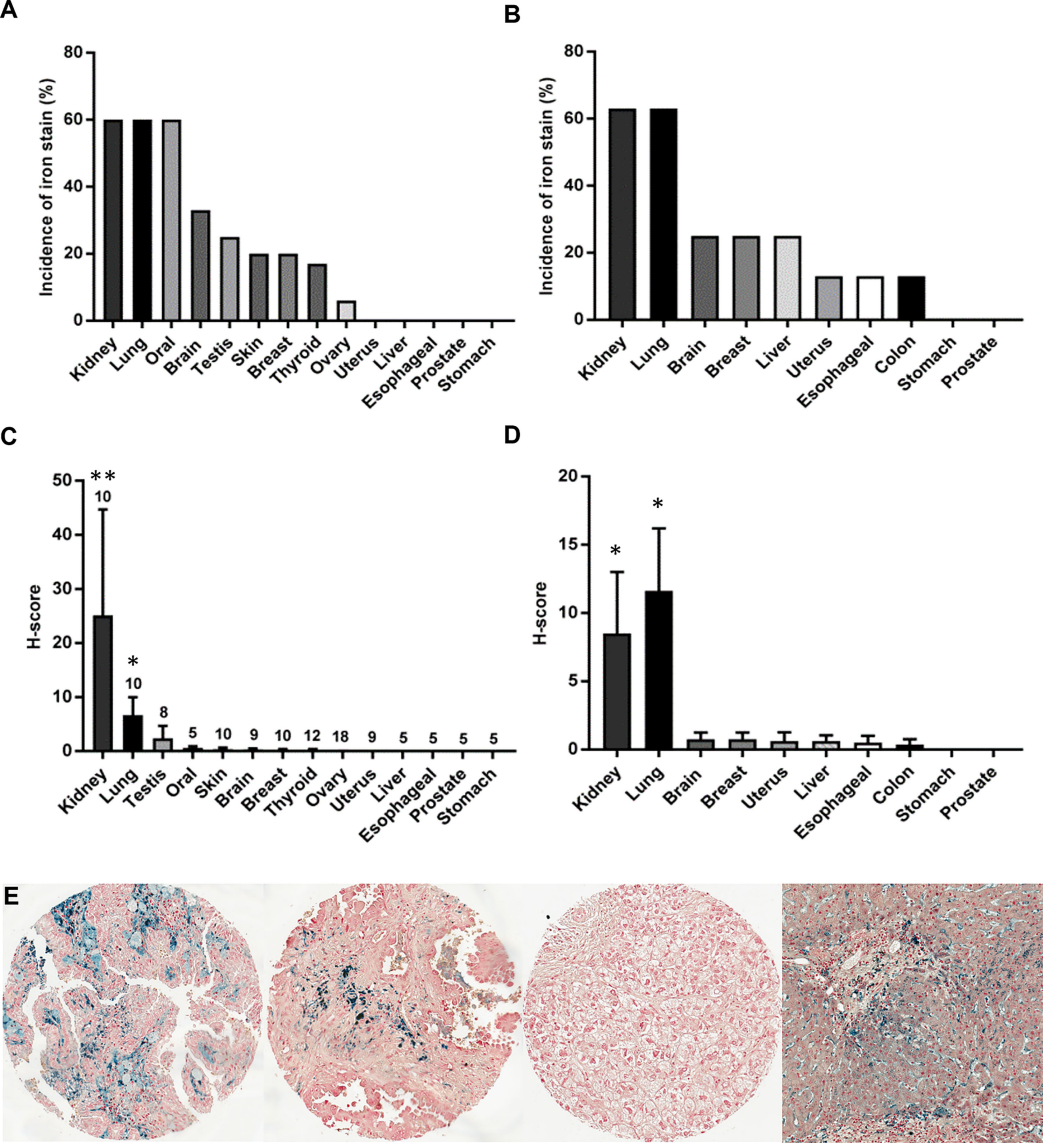
Kidney cancer has high levels of iron compared with other common cancers. The incidence and mean tissue level (H-score) of non-heme iron staining was compared across a variety of cancer types using two different TMA sources: **(A, C)** the RPCCC multi-cancer TMA that included primary tumor tissue from 14 different body sites and **(B, D)** the Biomax multi-cancer TMA that included primary tumor tissue from 10 different body sites. The number of patients evaluated for each tumor type in the RPCCC TMA is indicated above the error bar, and eight patients were evaluated for each tumor type in the Biomax TMA. **(E)** Representative staining images are shown at low power magnification for (left to right) kidney cancer (RCC), lung cancer, liver cancer, and hemochromatosis liver as a positive staining control for iron overload. *p < 0.01 *vs*. all other cancers; **p < 0.001 *vs*. all other cancers.

To compare tissue iron levels across various cancer types, non-heme iron was measured in 201 patient cancers from 15 different organ sites using Prussian Blue staining of two multi-cancer TMAs: 1) the RPCCC patient TMA that included 121 solid tumors from 14 different organ sites (**Figures 1A**, **C** **)**; and 2) the Biomax TMA that included 80 solid tumors from 10 different organ sites (**Figures 1B**, **D** **)**. Focal cellular iron accumulation was detected in most kidney (RCC), lung, and oral cancers, but only infrequently or not at all in other cancers (**Figures 1A**, **B**). Iron staining typically appeared as cytoplasmic punctate blue granules characteristic of Prussian Blue stain; however, some cells in kidney and lung cancers stained so intensely that the entire cell was dark blue and of greater stain intensity than observed in the liver iron-overload (positive control) tissue (**Figure 1E**). Kidney cancer had the highest mean iron staining score among all cancers in the RPCCC TMA (**Figure 1C**) and the second highest mean iron staining score (to lung cancer) among all cancers in the Biomax TMA (**Figure 1D**). Mean iron levels in kidney and lung cancers were each significantly higher than mean iron levels of all other cancers collectively (**Figures 1C**, **D**). Kidney and lung cancer iron staining was also common in the TME, particularly in regions of positively stained cancer cells. These results reveal that kidney cancer has high iron levels relative to other common cancers.

### Comparison of iron levels among primary tumors, metastases, and non-neoplastic kidney tissues of patients with RCC

**Figure 2:**
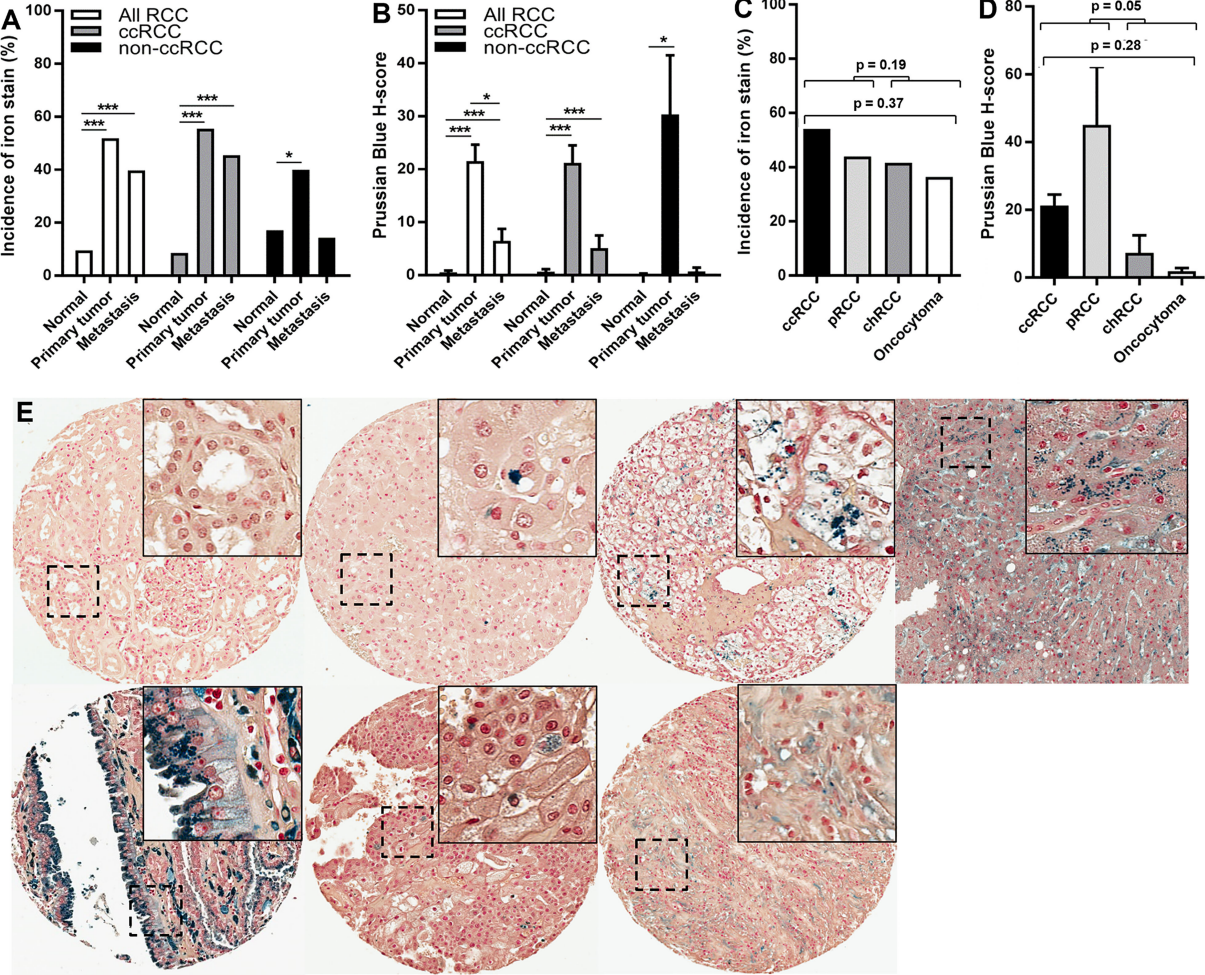
Increased iron levels in primary renal tumors and metastases. Prussian Blue staining for total iron was performed using the RPCCC RCC patient TMA set. **(A)** Iron staining incidence was compared among normal (non-neoplastic) kidney, renal primary tumors, and RCC metastases. **(B)** Mean iron levels (H-score) were compared among normal (non-neoplastic) kidney, primary tumors, and RCC metastases. **(C)** Iron staining incidence was compared among renal cell primary tumor histologic subtypes. **(D)** Mean iron levels (H-score) were compared among renal cell primary tumor histologic subtypes. **(E)** Representative images of iron staining from different kidney tissue types from left to right; top row: normal (non-neoplastic) kidney, renal oncocytoma, ccRCC, and hemochromotosis liver (positive control stain for iron overload); bottom row: papillary RCC, chromophobe RCC, and ccRCC metastasis. *p < 0.05; ***p < 0.001.

To more deeply interrogate kidney cancer iron content, tissue iron levels were measured using Prussian Blue stain in a large RCC patient TMA set harboring over 1,400 evaluable tissue cores from 570 tissue specimens (266 primary tumors, 231 normal/non-neoplastic kidney tissues, and 73 metastases) of 286 renal cell tumor patients (median/mean = 3.0/2.5 evaluable cores per specimen). Clinicopathologic features of these patients and their tissues are summarized in **Supplementary Table 1**. Detectable iron staining in renal epithelium of needle-core sections from non-neoplastic kidney tissue was uncommon (22 of 231 patients, 9.5%) and always focal in nature (**Figures 2A**, **E**). In contrast, tumor cell iron staining was detected in needle-core sections of most primary renal tumors (138 of 266 patients, 51.9%; p < 0.001) and was typically focal but occasionally diffuse, as in the liver iron-overload (positive control) tissue stain (**Figures 2A**, **E**). Highest staining incidence by histologic subtype was observed with ccRCC followed closely by pRCC (**Figure 2C**). As observed with the multi-cancer TMAs, positively stained renal tumor cells in the RPCCC TMA set frequently had staining intensity beyond that of liver iron-overload control tissue. Mean iron staining scores were on average over 40-fold higher in primary tumor cells compared with benign renal tubule epithelium (mean H-score = 21.6 *vs*. 0.5, respectively; p < 0.001) (**Figure 2B**). Mean iron levels in pRCC and ccRCC were significantly higher than mean iron levels in chRCC and benign renal oncocytoma (**Figures 2D**, **E**), with pRCC tending to have the highest levels but also the greatest variation.

As with primary tumors, metastasis tissues showed a higher incidence (29 of 73 cases, 39.7%) and a higher mean level (H-score = 6.5) of iron staining compared with non-neoplastic renal epithelium (p < 0.001 each) (**Figures 2A**, **B**). However, iron staining scores in metastases were on average more than three times lower than iron staining scores in primary tumors (**Figure 2B**). Similarly, iron staining scores in metastatic primary tumors were on average more than three times lower than iron staining scores in non-metastatic primary tumors (**Table 1**). Iron levels in metastases were not significantly different from iron levels in metastatic primary tumors (**Supplementary Table 2** **).** Collectively, these data indicate that total iron levels increase dramatically in primary tumors relative to non-neoplastic renal epithelium but then partially decline in metastatic primary tumors and their metastases.

**Table 1:** Association between iron levels in primary tumor cancer cells and clinical features of patients with RCC.

		Tumor Iron Level (Mean H-Score/SE)		
		All RCC	ccRCC	Non-ccRCC
Age, years	≤ 60 >60 p-value	18.6/3.827.1/5.40.23	21.3/4.521.1/4.90.89	13.1/8.853.8/22.6**0.027**
Gender	MaleFemaleP-value	27.1/4.515.2/4.00.055	26.4/4.713.1/4**0.025**	34.9/15.622/13.80.86
Race	CaucasionAAp-value	23.1/3.410.4/5.20.70	21.8/3.42.2/1.60.38	34.7/13.49.8/6.11.00
Body mass index, kg/m^2^	<30>= 30p-value	30.1/5.916.6/3.70.32	26/5.616.7/40.55	50.2/22.118.2/10.70.30
Smoking history	NeverAnyp-value	17.5/3.926.2/4.90.17	18.1/4.424.8/4.90.22	15.5/10.243.8/18.90.12
Pack years	0≤ 30>30p-value	17.5/3.922.8/5.831.5/10.90.47	18.1/4.425/6.928.5/10.70.61	15.5/10.214.7/8.653.7/46.60.48
Iron supplementation	NoYesp-value	26.1/3.92.2/1.20.35	23.3/3.92.6/1.50.51	36/13.30/00.26
Anemia	NoYesp-value	32.8/6.914.2/5.90.085	29.4/7.29.7/4.70.065	46.2/20.931.1/21.70.84
Microcytic anemia	NoYesp-value	31.8/6.21.7/1.20.13	28.7/6.42/1.40.17	43.9/17.20/00.29
Hemoglobin, g/dl	< 13.3≥ 13.3p-value	14.4/5.435.3/7.7**0.037**	11.7/4.930.4/7.70.084	26.6/18.752.3/23.30.44
Hypertension	NoYesp-value	30.2/7.421.6/4.30.36	20.9/5.421.9/50.85	77.2/3414.8/8**0.041**
Presence of metastasis	NoYesp-value	25.9/3.87/3.3**0.005**	24.3/3.98.4/3.9**0.012**	32.8/120/00.17

AA, African American; SE, standard error. Bold p-values signify statistical significance.

### Relation of RCC cellular iron levels to patient clinical features, tumor pathology, and survival

RCC cell iron levels (Prussian Blue H-score) in the RPCCC RCC patient TMA set were tested for association with patient features, including diagnostic and prognostic clinical risk factors **(** **Table 1**). Male patients with RCC had nearly twice as high cancer cell iron staining scores as female patients with RCC (p = 0.055), an association which reached significance in the ccRCC subset (p = 0.025). RCC cell iron levels were significantly associated with serum hemoglobin and, in the non-ccRCC patient subset, with older patient age.

**Figure 3:**
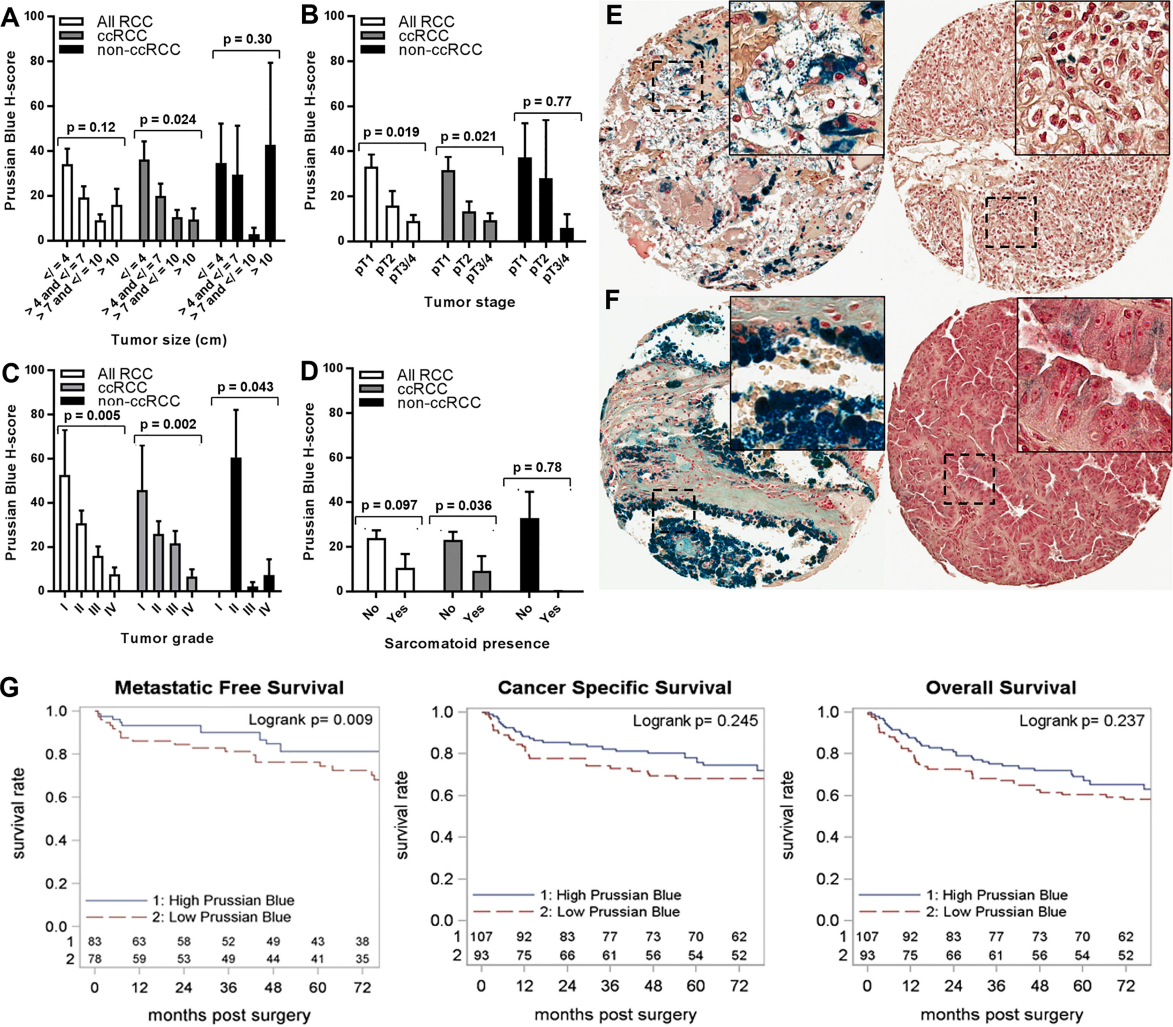
Iron levels in primary tumors decrease with tumor progression. Primary tumor iron levels (H-score) were evaluated using Prussian Blue stain of the RPCCC RCC patient TMA set and tested for association with pathologic features of primary tumors including **(A)** tumor size (largest diameter), **(B)** tumor stage, **(C)** tumor grade, and **(D)** presence of sarcomatoid dedifferentiation. Representative tissue core images are shown for low stage/grade (left) and high grade/stage (right) primary tumors of patients with **(E)** ccRCC and **(F)** pRCC. **(G)** Iron staining level (H-score) for the ccRCC patient subset was dichotomized at the median and tested for association with (left to right) metastatic-free survival, cancer-specific survival, and overall survival using Kaplan–Meier methodology.

RCC cell iron levels were also tested for association with key prognostic pathology of the primary tumor (**Figures 3A**–**F**). Lower RCC cell iron was significantly associated with worse pathological stage and grade (**Figures 3B**, **C**), whereas similar associations with larger tumor size (p = 0.12) and sarcomatoid dedifferentiation (p = 0.097) did not reach statistical significance (**Figures 3A**, **D**). In patients with the ccRCC subtype, these patterns were more pronounced, as lower iron was significantly associated with all major adverse pathological variables, including tumor size, stage, grade, and sarcomatoid dedifferentiation (**Figures 3A**–**E**). Non-ccRCC subset analyses were limited by greater staining variability and a smaller sample size but nonetheless revealed a similar significant association between lower cancer cell iron and higher tumor grade (**Figures 3C**, **F**). Together, these data indicate that RCC cell iron in primary tumors decreases with pathological progression, particularly for the ccRCC subtype, which is consistent with the lower RCC iron levels observed in metastatic primary tumors.

We next evaluated the relationship between RCC cell iron and metastasis-free, cancer-specific, and overall patient survival. Among patients with ccRCC, lower primary tumor cell iron was associated with significantly shorter metastasis-free survival (p = 0.013) but was not associated with cancer-specific survival (p = 0.17) or overall survival (p = 0.21) (**Table 2**; **Figure 3G**). In multivariable analyses, ccRCC iron levels did not independently predict worse metastasis-free survival after adjusting for tumor size, grade, and stage (p = 0.24) (**Table 2**). In the non-ccRCC patient subset, cancer cell iron levels were not significantly associated with survival outcomes (**Table 2**, **Supplementary Figure 1A**).

**Table 2:** Association between tissue iron levels and RCC patient survival outcomes.

Tissue site		Time to Metastasis				Time to Cancer-Specific Mortality				Time to All-Cause Mortality			
		Univariate		Multivariable		Univariate		Multivariable		Univariate		Multivariable	
		HR (95% CI)	p-value	HR (95% CI)	p-value	HR (95% CI)	p-value	HR (95% CI)	p-value	HR (95% CI)	p-value	HR (95% CI)	p-value
ccRCC patients	Tumor Cancer Cells	0.45 (0.23, 0.87)	**0.013**	0.63 (0.29, 1.37)	0.24	0.70 (0.42, 1.17)	0.17	–	–	0.77 (0.51, 1.16)	0.21	–	–
	TME	0.54 (0.27, 1.09)	0.068	–	–	0.84 (0.49, 1.42)	0.48	–	–	0.66 (0.43, 1.02)	0.053	–	–
	Benign renal epithelium	3.02 (1.27, 7.17)	**<0.001**	4.95 (1.65, 14.88)	**0.004**	3.46 (1.74, 6.89)	**<0.001**	1.71 (0.78, 3.75)	0.18	2.17 (1.12, 4.17)	**0.028**	1.12 (0.53, 2.37)	0.76
non-ccRCC patients	Tumor Cancer Cells	0.45 (0.10, 2.06)	0.22	–	–	0.65 (0.13, 3.21)	0.50	–	–	2.18 (0.77, 6.11)	0.12	–	–
	TME	0.50 (0.09, 2.65)	0.34	–	–	0.71 (0.16, 3.17)	0.62	–	–	1.90 (0.65, 5.54)	0.20	–	–
	Benign renal epithelium	1.82 (0.24, 14.07	0.78	–	–	1.04 (0.16, 6.96	0.79	–	–	1.57 (0.45, 5.46)	0.60	–	–

Hazard ratios (HR) refer to iron levels (H-scores) above the median. Multivariable analysis was performed only if time to event was significant on univariate analysis. CI, confidence interval; TME, tumor microenvironment. Bold p-values signify statistical significance.

### Tumor microenvironment iron levels and relation to RCC patient features and survival

Iron staining levels were also evaluated within the TME and benign renal cortical stroma using the RPCCC RCC patient TMA set. Foci of iron stain were commonly detected in the TME including stroma of primary tumors (108 of 266 cases, 40.6%) and metastases (27 of 73 cases, 37.0%) but rarely in the stroma of non-neoplastic kidneys (18 of 231 cases, 7.8%, p < 0.001 each). Microenvironment/stromal iron staining scores were on average 24-fold higher in primary tumors (mean H-score = 9.4) and 10-fold higher in metastases (mean H-score = 4.1) than in non-neoplastic kidneys (mean H-score = 0.4, p < 0.001 each). The mean microenviroment iron staining score of metastases was less than half that of primary tumors, but this difference was not statistically significant (p = 0.22). There was a significant strong correlation by Spearman testing between cancer cell iron levels and microenvironment iron levels within the same primary tumor or metastasis; and a significant moderate correlation between renal epithelial iron levels and renal stromal iron levels within the same kidney (**Table 3**). Histologic subtype differences in primary TME iron levels mirrored subtype differences observed in cancer cell iron levels, with pRCC and ccRCC having highest levels (mean H-scores: pRCC 11.3, ccRCC 10.5, chRCC 5.6, and oncocytoma 1.2), although these differences were not statistically significant (p = 0.22).

**Table 3:** Spearman correlation of iron levels in the tumor microenvironment (TME)/stroma versus epithelium of the same RCC patient tissue specimen.

Tissue site	Epithelial Iron *vs*. TME/Stromal Iron (H-Score)					
	All RCC		ccRCC		Non-ccRCC	
	Spearman coefficient	p-value	Spearman coefficient	p-value	Spearman coefficient	p-value
Primary tumor	0.64	**<0.001**	0.62	**<0.001**	0.81	**<0.001**
Benign kidney	0.47	**<0.001**	0.43	**<0.001**	0.69	**<0.001**
Metastasis	0.65	**<0.001**	0.66	**<0.001**	1.0	**<0.001**

Bold p-values signify statistical significance.

**Figure 4:**
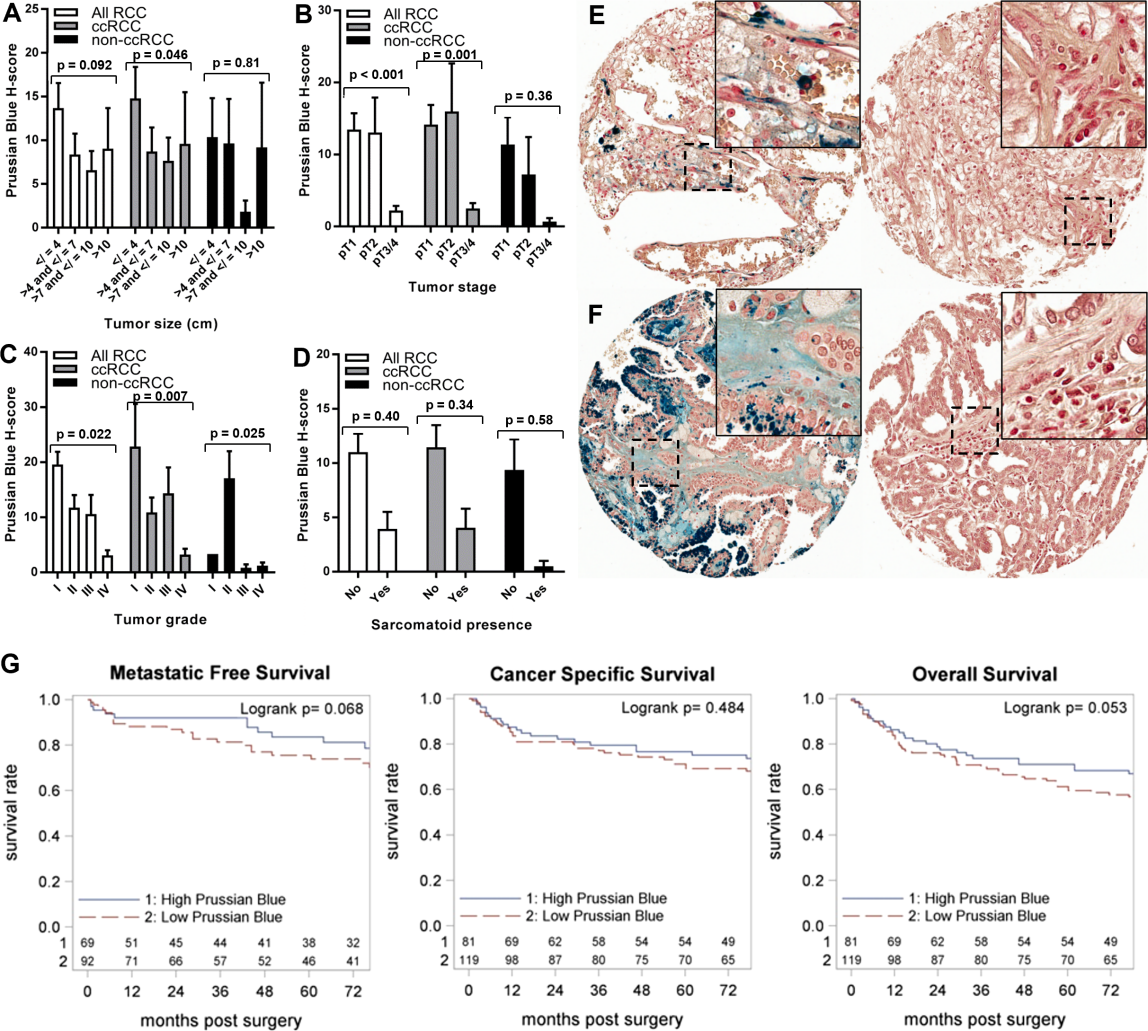
Iron levels within the tumor microenvironment decrease with tumor progression. Tumor microenvironment iron levels (H-score) were evaluated using Prussian Blue stain of the RPCCC RCC patient TMA and tested for association with pathologic features of primary tumors including **(A)** tumor size (largest diameter), **(B)** tumor stage, **(C)** tumor grade, and **(D)** presence of sarcomatoid dedifferentiation. Representative tissue core images of tumor microenvironment iron staining are shown for low stage/grade (left) and high grade/stage (right) primary tumors of patients with **(E)** ccRCC and **(F)** pRCC. **(G)** Iron staining level (H-score) for the ccRCC patient subset was dichotomized at the median and tested for association with (left to right) metastatic-free survival, cancer-specific survival, and overall survival using Kaplan–Meier methodology.

Primary TME iron level was tested for association with RCC patient clinicopathologic features and survival outcomes (**Tables 2**, **4**; **Figure 4**). Primary TME iron level was not associated with any clinical risk factors in the overall RCC patient cohort (**Table 4**) but significantly declined with pathological progression in tumor stage (p < 0.001) and grade (p = 0.022) (**Figures 4B**, **C**), mirroring patterns observed in cancer cell iron levels. An association between lower RCC TME iron and metastatic stage approached significance (p = 0.058) (**Table 4**). In the ccRCC patient subset, lower TME iron levels were significantly associated with patient anemia and worse tumor size, stage, and grade (**Figures 4A**–**C**). An association between lower ccRCC TME iron levels and shorter time to metastasis approached significance (p = 0.068) (**Table 2**; **Figure 4G**) and reached significance (HR = 0.59, p = 0.023) if patients with metastases at the time of surgery were included. An association between lower ccRCC microenvironment iron and all-cause mortality also approached significance (p = 0.053) (**Table 2**; **Figure 4G**). In non-ccRCC patients, there was a significant association between lower TME iron levels and higher tumor grade (**Figure 4C**) but no associations with survival outcomes (**Table 2**; **Supplementary Figure 1B**). Collectively, these data indicate that the TME iron level decreases with RCC pathologic progression to metastasis, particularly for ccRCC, which mirrors associations observed in the cancer cell compartment.

**Table 4:** Association between tumor microenvironment iron levels and RCC patient clinical features.

		Iron Stain Level (Mean H-Score/SE)		
		All RCC	ccRCC	Non-ccRCC
Age, years	≤ 60>60P-value	3.4/0.74.4/0.90.32	4/0.93.9/0.90.82	1.9/0.98.2/3.40.11
Gender	MaleFemaleP-value	4.2/0.73.3/0.90.25	3.9/0.84/1.10.71	6.4/2.41.2/0.50.29
Race	CaucasianAAP-value	3.8/0.65.1/3.40.42	3.8/0.69.3/7.90.49	5.2/1.91.7/1.20.56
Body mass index, kg/m^2^	<30≥ 30P-value	3.6/0.84.5/1.00.67	3.1/0.84.7/1.10.99	6.7/3.13.7/1.70.33
Smoking history	NeverAnyP-value	4/0.93.9/0.80.89	4.6/1.13.5/0.80.98	2/0.86.9/2.90.46
Smoking pack years	0≤ 30>30P-value	4/0.93.4/0.94.3/1.50.92	4.6/1.13.6/1.24.4/1.70.91	2/0.81.9/1.04.9/4.10.93
Iron supplementation	NoYesP-value	4.5/0.71.3/0.70.33	4.3/0.81.7/0.80.81	5.5/1.90/00.19
Anemia	NoYesP-value	6.1/1.22.6/1.10.077	6.3/1.51.4/0.5**0.047**	5.9/2.15.9/4.40.74
Microcytic anemia	NoYesP-value	5.7/1.11/0.60.18	5.5/1.31/0.70.19	6.6/2.50.8/0.80.76
Hemoglobin, g/dl	< 13.3≥ 13.3P-value	3.9/1.35.4/1.20.24	3.6/1.35.1/1.40.45	5.1/3.86.7/2.30.32
Hypertension	NoYesP-value	4.5/1.24.2/0.8.78	3.5/1.04.5/1.0.60	9.5/5.03.3/1.3.11
Presence of metastasis	NoYesP-value	4.4/0.71.5/0.60.058	4.4/0.81.7/0.70.17	5/1.70.2/0.20.35

AA, African American; SE, standard error.

### Relation of non-neoplastic renal epithelial iron levels to RCC patient features and survival

**Figure 5:**
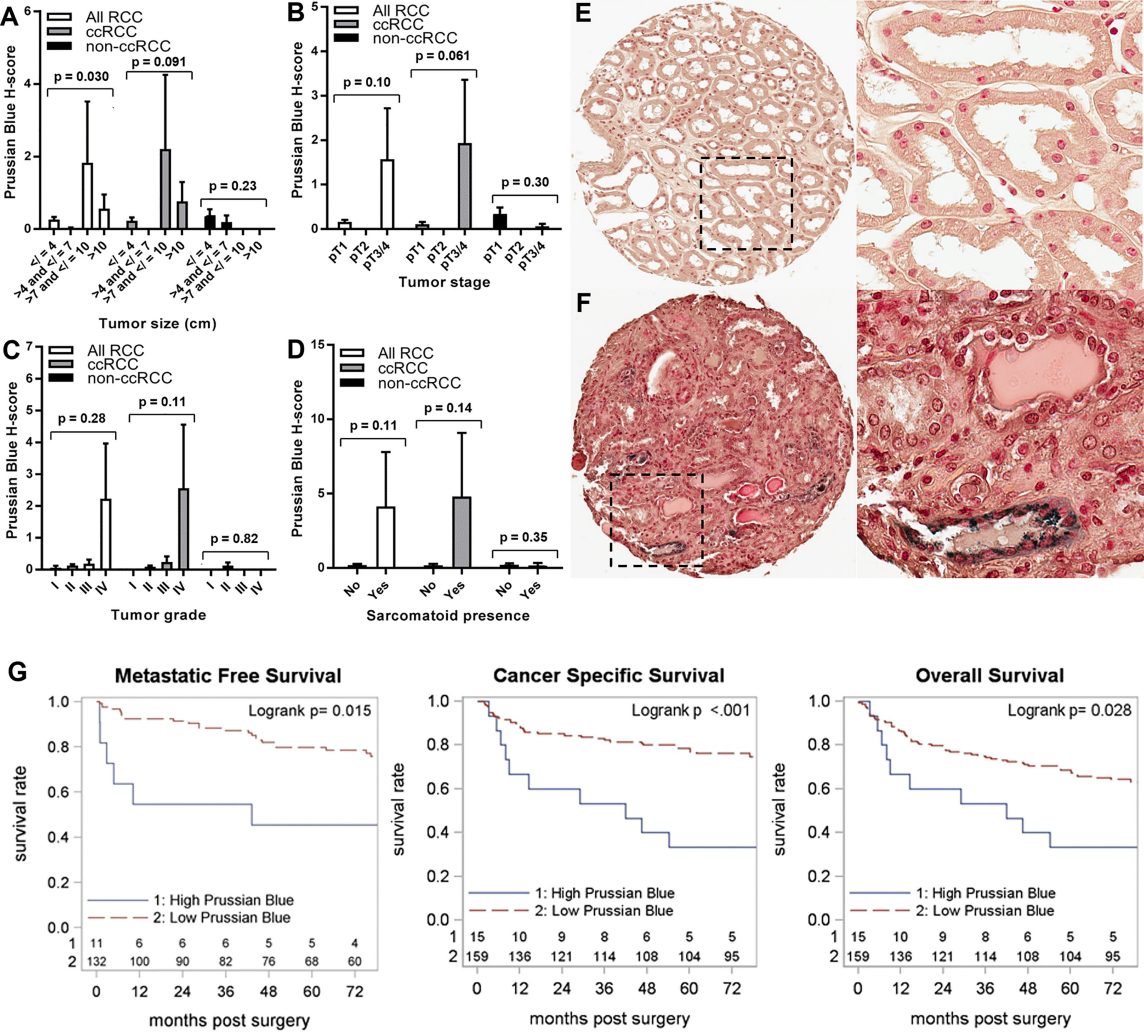
Iron levels increase in non-neoplastic kidney during tumor progression. Normal (non-neoplastic) renal tubule epithelial iron levels (H-score) were evaluated using Prussian Blue stain of the RPCCC RCC patient TMA set and tested for association with pathologic features of primary tumors including **(A)** tumor size (largest diameter), **(B)** tumor stage, **(C)** tumor grade, and **(D)** presence of sarcomatoid dedifferentiation. Representative tissue core images of normal kidney tissues are shown at low magnification (left) and high magnification (right) for a patient with **(E)** low grade RCC and a patient with **(F)** high grade RCC. **(G)** Iron staining level (H-score) for the ccRCC patient subset was tested for association with (left to right) metastatic-free survival, cancer-specific survival, and overall survival using Kaplan–Meier methodology.

We also examined the relationship of adjacent non-neoplastic renal epithelial iron levels to RCC patient clinicopathologic features and survival outcomes (**Table 5**; **Figure 5**; **Supplementary Figure 1C**). Significantly higher renal epithelial iron levels were detected in older RCC patients (**Table 5**). Precisely opposite to patterns in cancer cells, renal epithelial iron levels significantly increased with worsening RCC pathology, including size, stage, grade, and sarcomatoid dedifferentiation (**Figures 5A**–**F**). Among ccRCC (but not non-ccRCC) patients, higher renal epithelial iron was strongly associated with shorter times to metastasis (p < 0.001), cancer-specific death (p < 0.001), and death due to any cause (p = 0.028) (**Figure 5G**; **Table 2**; **Supplementary Figure 1C**). In multivariable analyses adjusting for primary tumor pathology, renal epithelial iron levels in patients with ccRCC remained independently associated with metastasis-free survival (HR = 4.95, p = 0.004) but not cancer-specific survival (HR = 1.71, p = 0.18) or overall survival (HR = 1.12, p = 0.76) (**Table 2**).

**Table 5:** Association between benign renal epithelial iron levels and RCC patient clinical features.

		Benign renal epithelium iron level (mean H-Score/SE)		
		All RCC	ccRCC	Non-ccRCC
Age, years	≤ 60>60p-value	0.2/0.11/0.7**0.013**	0.3/0.11.1/0.90.33	0/00.4/0.2**0.007**
Gender	MaleFemalep-value	0.2/0.11.2/0.90.58	0.2/0.11.6/1.20.73	0.3/0.10/0**0.045**
Race	CaucasianAAp-value	0.5/0.41.1/1.0.38	0.6/0.52.3/2.30.31	0.2/0.10.3/0.21.00
Body mass index, kg/m^2^	<30≥ 30p-value	0.2/0.11.2/0.80.32	0.2/0.11.3/1.00.27	0.2/0.10.3/0.20.82
Smoking history	NeverAnyp-value	0.9/0.80.3/0.10.13	1.1/1.00.4/0.20.15	0.2/0.10.2/0.10.48
Smoking pack years	0≤ 30>30p-value	0.9/0.80.3/0.20.4/0.30.42	1.1/1.00.4/0.20.5/0.40.39	0.2/0.10.2/0.10/00.63
Iron supplementation	NoYesp-value	0.7/0.50.3/0.3.92	0.8/0.60.4/0.40.64	0.3/0.10/00.38
Anemia	NoYesp-value	0.4/0.21.8/1.60.80	0.4/0.22.3/2.10.99	0.4/0.20.1/0.10.46
Microcytic anemia	NoYesp-value	0.3/0.10.1/0.10.72	0.4/0.20.1/0.10.88	0.3/0.10/00.46
Hemoglobin, g/dl	< 13.3≥ 13.3p-value	1.5/1.40.4/0.20.75	1.9/1.80.4/0.30.65	0.2/0.10.4/0.20.86
Hypertension	NoYesp-value	1.3/1.30.3/0.10.22	1.6/1.60.3/0.2.55	0/00.4/0.2.24
Presence of metastasis	NoYesp-value	0.6/0.40.4/0.20.89	0.7/0.50.4/0.30.36	0.2/0.10/00.45

AA, African American; SE, standard error. Bold p-values signify statistical significance.

### Relation of tissue iron to TfR1 protein levels in patients with RCC

The same renal tumor tissues undergoing iron staining also previously underwent immunohistochemical staining for the main iron uptake receptor, TfR1, as separately reported (28). Here, we tested whether tissue iron levels correlate with tissue TfR1 protein levels from the same patient (**Table 6**). In RCC cells of primary tumors, a significant negative correlation of weak strength was observed between iron levels and TfR1 levels. In RCC cells of metastases, a significant negative correlation of moderate strength was observed between iron and TfR1 levels. In non-neoplastic renal epithelium adjacent to the primary tumor, no correlation between iron and TfR1 levels was observed, although this analysis was limited by very infrequent iron detection.

**Table 6:** Spearman correlation of tissue iron levels versus TfR1 protein levels in RCC patients.

Tissue Type	Iron Level *vs*. TfR1 Level (H-Score)					
	All Patients		ccRCC		Non-ccRCC	
	Spearman coefficient	p-value	Spearman coefficient	p-value	Spearman coefficient	p-value
Benign renal epithelium	−0.04	0.53	−0.08	0.29	0.01	0.94
Primary tumor (cancer cell)	−0.21	**<0.001**	−0.23	**0.0013**	0.11	0.50
Metastasis (cancer cell)	−0.30	**0.0088**	−0.44	**0.010**	0.61	0.14

Bold p-values signify statistical significance.

## Discussion

Accumulating data support a unique role for iron in RCC pathogenesis but with potentially different mechanistic contributions to carcinogenesis, tumorigenesis, and progression (40–42, 45–47, 64, 65, 67). The recent discovery of ferroptosis resistance as a potentially important therapeutic target in advanced RCC suggests that iron and its oxidative stress effects, historically thought to be tumor promoting, might also serve as a liability for progressing cancers (39, 55–58). The fundamental question of tissue iron levels and their dynamics during RCC tumorigenesis and progression thus has relevance for novel therapeutic strategy development.

To our knowledge, the current study represents the most extensive investigation of tissue iron levels and their prognostic significance in patients with RCC. We discovered that RCC primary tumors have dramatically higher total iron content relative to non-neoplastic renal epithelium and most other common solid cancers, with accumulation arising in both the cancer cell compartment and TME. Unexpectedly, RCC iron content decreased significantly with pathological progression and metastasis, particularly for the ccRCC subtype, predicting significantly worse metastasis-free patient survival, despite precisely opposite iron changes occurring simultaneously in adjacent renal epithelium. Patients with large tumors (>7 cm), pT3-pT4 stage, or grade IV/sarcomatoid dedifferentiation had lowest tumor iron and highest renal epithelial iron. These results support a novel model for RCC, particularly ccRCC, in which tumor iron accumulates with early tumorigenesis but decreases with later progression despite concurrent increases in adjacent renal epithelial iron, suggesting distinct tissue-specific roles for iron during carcinogenesis and early tumorigenesis *vs*. later progression. Although non-ccRCC analyses in this study were limited by a smaller cohort with greater variability in tumor iron content, the significant inverse association between non-ccRCC iron levels and tumor grade suggests that a similar model might apply to non-ccRCC subtypes as well.

We also observed a significant inverse correlation between levels of iron and TfR1 protein within the same tumor, which indicates that iron level dynamics during RCC progression are likely a driver of, rather than a response to, alterations in TfR1 levels (28). TfR1 elevation is a well-described feedback response to low cellular reactive iron (25, 26), and our previous finding of lower TfR1 expression in ccRCC and pRCC tumors relative to chRCC tumors (28) may reflect the higher iron content of ccRCC and pRCC tumors relative to chRCC tumors suggested by the current study. Progressive loss of iron despite increasing TfR1 overexpression in pathologically adverse primary tumors (but not adjacent kidney tissues) thus suggests an increasingly iron-deficient tumor state in patients with advanced RCC. An important question raised by this work is what proportion of the tumor cellular iron content is free (i.e., reactive) *vs*. bound (i.e., inert) in ferritin or endocytosed transferrin. Although future investigation is needed, we expect that RCC free iron levels will mirror the total iron levels detected in the current study, because feedback changes in TfR1 protein levels are driven by levels of free rather than stored iron.

Iron has long been implicated in carcinogenesis based on animal modeling experiments, epidemiologic observations, and a clinical trial that significantly reduced cancer diagnoses in patients randomized to serum iron reduction (39–47). However, tissue iron levels in different cancers and their prognostic significance have undergone little investigation to date. To our knowledge, the current study is the largest to evaluate tumor iron levels for any cancer type and also the first to compare iron levels among a broad variety of cancers. In contrast to our findings in patients with RCC, Prussian Blue staining in hepatocellular carcinoma patients revealed only low or absent tumor iron deposits relative to non-neoplastic liver (78–81). Tumor iron deposits were also not detectable in studies of patients with papillary thyroid cancer, melanotic schwannoma, and basal cell carcinoma (82–84). On the other hand, Alwahaibi et al. identified tissue iron deposits in 48% of patient lung cancers (85). Prior studies of RCC iron levels have been limited to smaller patient cohorts but collectively support the common presence (27%–76%) of tumor iron accumulation (86–89), with MRI-based detection additionally suggesting a similar incidence between ccRCC and pRCC (90). Altogether, this prior literature supports our conclusion that iron accumulation is common in RCC (especially ccRCC and pRCC) and lung cancer but not in other solid cancers.

The cause of iron accumulation with RCC tumorigenesis is unclear. Potent ROS generated by iron reaction with hydrogen peroxide may have carcinogenic potential (22–24, 39, 51), and iron may also promote cell proliferation as a DNA synthesis cofactor and indirect regulator of cell cycle proteins such as p53, Rb, p21, and p27 (48–50). However, these mechanisms alone would not explain tumorigenic specificity for kidney tissue (39–42, 45–47). In that regard, we and others have observed that master proteins for cellular free iron elevation (TfR1, DMT1/SLC11A2, and IRP1/ACO1) collectively have highest body levels in renal tubule epithelium (28–30), perhaps making this tissue prone to iron accumulation and oxidative stress injury (91). In rodents, repeated high dosing of iron chelated to nitrilotriacetic acid (FeNTA model) effectively targets iron deposition to the renal epithelium, which induces RCC tumorigenesis with high frequency (40–42, 92). In humans, multiple medical conditions with high serum iron that also trigger iron deposition specifically in the kidney (e.g., sickle cell disease) have significantly higher risk for kidney cancer development (64–66). Iron deposition specifically to renal epithelium is also induced by hypertension, diabetes, obesity, and chronic kidney disease, all of which are clinical risk factors for RCC (31–38). In contrast, neither hereditary iron overload (hemochromatosis) nor dietary iron overload induces renal iron deposition nor increases the risk for RCC diagnosis (66). Reported links of RCC diagnosis with iron industry occupations and a micro-RNA binding site polymorphism in the *TFRC* (TfR1) gene remain of unknown clinical significance (45–47, 67), as does the presence of abundant iron in the RCC carcinogen, tobacco (93, 94).

The unique cancer genetics of ccRCC may contribute to the distinctive iron accumulation that typifies this cancer, particularly in its early stage. We recently described that VHL genetic loss in ccRCC cells introduces a cell dependency on free iron for escape from apoptosis and cell cycle arrest (74). We also found reactive iron to be significantly higher in VHL-inactivated ccRCC cell lines relative to non-neoplastic renal epithelial cell lines (74), similar to the differences in total tissue iron observed in the current study. Mechanisms underlying iron dependency in VHL-inactivated tumors are unclear but might relate to the requirement of reactive iron for translation of HIF-2α, the primary oncoprotein driver for VHL-inactivated ccRCC tumorigenesis, due to a rare iron response element (IRE) in the HIF-2α transcript (71). HIF-2α protein is also a direct transcriptional activator of several master genes for cellular iron elevation including *TFRC* and *SLC11A2* (72, 73), which raises the possibility of cooperative feed-forward accumulation between iron and HIF-2α upon VHL inactivation (74). However, this hypothesis cannot account for tumor iron accumulation in non-ccRCC subtypes, which lack HIF-2α overexpression. Alternatively, iron dependency of ccRCC to avoid cell cycle arrest and apoptosis may be unrelated to HIF-α, similar to the recent demonstration that VHL loss introduces a novel requirement for cell cycle kinases (CDK4/6) in a HIF-α–independent manner (95).

Given evidence of iron accumulation in RCC tumors and the conventional understanding of iron’s role in carcinogenesis (39, 54, 57), our additional discovery of tumor iron reduction with RCC progression is unexpected and paradigm-challenging. To our knowledge, this finding is unique for any cancer studied to date. In contrast to this finding, Jamnongkan et al. and de la Monte et al. previously reported that lower tumor iron detected with Prussian Blue stain predicted better rather than worse oncologic outcomes for cholangiocarcinoma patients and meningioma patients, respectively (96, 97); whereas Tsuji et al. identified no association between tumor iron staining and cancer recurrence in patients with hepatocellular carcinoma (98). Prior to contemporary RCC histologic subtype classifications, Delahunt et al. reported no prognostic significance of tissue iron deposits among 102 renal tumor patients, although almost all tumors evaluated were low grade (99), and a more recent study of six patients with an indolent pRCC variant detected abundant iron accumulation in all but one tumor (87). Different conclusions of Delahunt et al. and the current study may reflect our larger cohort with more widely represented pathology (size/grade/stage), updated subtype classifications, and broader scale for iron stain scoring.

The cause of tumor iron reduction with RCC progression requires further investigation. It is possible that iron’s potent oxidizing reactivity may become a hindrance to cancer cells by inducing ROS sufficient to trigger regulated cell death (39, 58–63). Partial iron reductions that avoid oxidative stress injury but still support cell proliferation may then become necessary. Resistance to iron-dependent oxidative stress–induced regulated cell death, or ferroptosis, is increasingly recognized as an important mechanism for progression in a variety of cancers (39, 55–58), particularly ccRCC, which is uniquely susceptible to ferroptosis targeting in preclinical models (75, 100). As with the heightened iron requirements of ccRCC, the heightened sensitivity of ccRCC to ferroptosis may relate to the unique genetics of this cancer. Indeed, VHL inactivation appears to predispose cells to ferroptosis induction (76), and additional alterations may be necessary for ccRCC cells to escape this event. Such alterations may include BAP1 loss (15% of all ccRCC tumors) that is associated with an aggressive ccRCC phenotype and promotes ferroptosis escape by increasing SLC7a11-mediated cysteine uptake for antioxidant production (101, 102). Ferroptosis escape can be induced experimentally by iron depletion (55); hence, lower tumor iron in patients with RCC might confer a selective advantage.

We thus suspect that the biphasic dynamics of iron levels with RCC initiation *vs*. progression may reflect differential responses to iron-induced oxidative stress. The dual role of oxidative stress in promoting early tumorigenesis/carcinogenesis yet suppressing later progression is increasingly recognized, invoking such nomenclature as “two-faced” and a “double-edged sword” (103–107). Physiologic ROS levels are a natural by-product of oxygen metabolism, but the initial agents generated in these reactions, including hydrogen peroxide, harbor only weak oxidative potential and are generally inadequate for oxidative stress induction (39). In contrast, in what is known as the Fenton Reaction, iron uniquely reacts with the hydrogen peroxide by-product of oxygen metabolism to produce the much more potent ROS, the hydroxyl radical, which directly mediates most oxidative stress damage in the human body. Thus, oxidative stress is intimately tied to cellular iron levels. Innate antioxidant mechanisms to avoid oxidative stress involve a variety of enzymes, such as superoxide dismutases, catalase, peroxiredoxins, thioredoxins, glutathione/glutathione peroxidase, and heme oxygenase (108, 109). At low levels, ROS can promote survival and proliferation, exploiting various mitogenic signaling pathways implicated in tumorigenesis (including in RCC), such as those involving phosphatase and tensin homologue (PTEN), phosphoinositide 3 (PI3)-kinase, and platelet-derived growth factor (PDGF) (108). However, higher ROS levels can overcome antioxidant defenses and induce oxidative damage (i.e., stress) to cellular lipids, proteins and nucleic acids, including mutagenic alterations that promote carcinogenesis (22–24). Moreover, oxidative stress beyond a certain threshold triggers regulated cell death including apoptosis *via* mitochondrial membrane depolarization and especially iron-dependent ferroptosis due to lethal accumulation of lipid peroxidation (39, 55). Because cancers have constant oxidative stress and may be more sensitive to ROS than normal cells (58, 110), they must adapt acutely (metabolic reprograming) or chronically (genetic/genomic reprogramming) to balance maintenance of pro-mitogenic ROS signals with avoidance of oxidative damage and cell suicide (111, 112). This challenge may be more substantial for aggressive cancers, due to higher ROS levels from increased metabolic activity, and a variety of mechanisms by which cancer may evolve to reduce ROS are described (108). This paradigm is exemplified by recent characterizations of the redox genomic landscape in RCC patient tumors, revealing robust antioxidant upregulation and low oxidative stress in aggressive RCC tumors, compared with high oxidative stress in indolent RCC subtypes such as chRCC or clear-cell pRCC (113–117).

While ferroptosis escape provides a possible explanation for tumor iron reduction with RCC progression, an alternative reason may instead or additionally relate to decreased iron availability to cancer cells during progression. Consistent with this possibility, we found lower tumor iron to be more common in patients with RCC with lower serum hemoglobin, an indicator of reduced iron availability in the circulation. These findings are consistent with the established fact that patients with advanced RCC are more likely to be anemic (i.e., low serum iron) (118). However, anemia of patients with advanced RCC cannot alone explain tissue iron dynamics in this study because adjacent renal epithelial iron simultaneously increased with RCC progression. Opposing iron changes in renal epithelium *vs*. the tumor during RCC progression underscore yet-to-be characterized differences in molecular pathways for benign versus malignant renal epithelial iron metabolism regulation. Moreover, iron reduction in advanced RCC tumors provides compelling support that anemia of advanced RCC is not caused by increased tumor iron sequestration, as previously hypothesized (119). Alternative explanations for advanced RCC patient anemia include increased tumor secretion of inflammatory cytokines that may trigger mobilization of serum iron into reticuloendothelial tissue storage sites (120).

In further regard to a potential role for extracellular iron availability in mediating RCC tumor iron dynamics, the positive correlation between cancer cell iron levels and microenvironment iron levels within the same tumor suggests that the TME might serve as an important iron reservoir for RCC cells. In the current study, TME iron was commonly detected in extracellular matrix and cells that were morphologically consistent with macrophages, a major tissue iron reservoir. The genomic profile of tumor-associated macrophages (TAMs) has been shown to predict iron efflux (121). Moreover, TAMs may deliver iron to tumor cells using lipocalin 2 (122), which is a known growth factor for renal epithelial cells (123, 124); and the main iron storage protein complex, ferritin, is uniquely secreted by both macrophages and renal proximal tubule cells (125). pRCC tumors are commonly characterized by a distinct abundance of TAMs, which might explain the very high iron levels observed by us and others in a subset of pRCC tumors (86, 87). An alternative source of TME iron can be hemolyzed erythrocytes that release iron-bound heme after intratumoral hemorrhage. Although Prussian Blue does not directly stain heme, it does stain iron products of heme degradation catalyzed by heme-oxygenase 1 (HMOX1), which can be overexpressed in RCC tumors (126).

A potential clinical application of these findings is the exploitation of tumor iron levels as a novel therapeutic target for patients with advanced RCC. Durable patient responses to standard-of-care RCC therapies that inhibit either immune checkpoint control or downstream angiogenesis effects of VHL inactivation remain limited, and novel targets are needed (9). Pharmacologic approaches to reduce RCC tumor iron are of promise, given the increased susceptibility of ccRCC cells to iron chelator drugs in preclinical models (74). The relative iron-deficient state of metastatic RCC primary tumors and their metastases might translate into increased responsiveness of patients with advanced RCC to iron deprivation therapy, particularly because TfR1 is already overexpressed in metastatic RCC, and further feedback elevation in response to iron depletion may therefore be limited (28). Several iron chelator drugs are already approved for clinical use in refractory systemic iron overload and are generally well tolerated by patients (127). Furthermore, there is precedent for an oncologic benefit of iron chelation as either an adjuvant or monotherapy in patients with neuroblastoma and patients with advanced HCC, respectively (128, 129). Selective reduction of RCC iron *via* targeting of TfR1 or other iron metabolism proteins may improve upon non-specific iron-chelating strategies, with efficacy already suggested in preclinical RCC models (28, 130).

Of additional clinical potential is leveraging high RCC iron levels to therapeutically overcome ferroptosis resistance. The clinical promise of targeting ferroptosis resistance was recently highlighted at the 2020 Kidney Cancer Research Summit meeting (9). Heightened ferroptosis susceptibility of ccRCC due to VHL loss has been effectively exploited in preclinical models using cysteine depletion to reverse ferroptosis-inhibiting effects of BAP1 loss (131). This work and other similar works suggest that cysteine homeostasis could be an effective clinical target (9). Directly targeting RCC iron levels as either monotherapy or an adjuvant to other ferroptosis-inducing approaches provides similar promise, given that RCC has high intracellular iron levels, which predicts high ferroptosis susceptibility (55). Intriguingly, autophagy induction appears to enhance ferroptosis by degrading inert iron stores (ferritin) into reactive free iron. The efficacy of autophagy drugs in patients with cancer (including rapamycin in patients with RCC) provides indirect support for clinically targeting iron levels, and the combination of this approach with direct ferroptosis induction has achieved promising results in preclinical RCC models (131, 132). The current study provides a foundation to guide future ferroptosis-targeting strategies and suggests that patients with early stage (e.g., small renal mass) RCC may also be candidates given their particularly high tumor iron content.

Finally, this study reveals that iron accumulation detected with Prussian Blue stain may have clinical value as a novel prognostic biomarker for patients with RCC, which is of critical need (9, 14). Prussian Blue staining is already clinically standardized and familiar to pathologists, given its long-time use in hemochromatosis patients (77). Our findings indicate that this approach provides robust prognostic information for patients with ccRCC, with lower tumor iron and higher renal epithelial iron each predicting significantly more aggressive disease including metastatic potential. Although Prussian Blue stain in tumors did not provide additional prognostic value beyond surgical pathology alone, it may aid common clinical scenarios in which a tumor biopsy tissue is available without surgical pathology (e.g., small renal mass patients considering surveillance; high-risk localized patients considering a neoadjuvant drug trial, etc.), particularly given that RCC biopsies do not reliably assess tumor grade (133). In contrast to tumor biomarkers, biomarkers derived from non-neoplastic renal epithelium remain scarcely explored for RCC patient risk stratification (28). Intriguingly, renal epithelial iron staining in this study provided strong independent prognostic value beyond surgical pathology alone. This finding is consistent with our prior discovery of independent strong prognostic value for renal epithelial TfR1 protein levels in ccRCC and non-ccRCC patients (28). Staining for iron and TfR1 protein in renal epithelium, in addition to other iron metabolism proteins, thus warrants future study for the clinical risk stratification of RCC nephrectomy patients [e.g., patients with clinically localized RCC considering adjuvant drug therapy (134)], as do the enigmatic mechanisms underlying their intriguing prognostic impact.

## Conclusion

A growing body of literature supports that iron has a unique role in RCC pathogenesis, with the recent discovery of ferroptosis resistance suggesting that iron’s potent oxidative reactivity, historically presumed to be tumor promoting, might also serve as a liability for cancer progression. The current study reveals that RCC tumors have atypically common iron accumulation relative to non-neoplastic kidney tissue and other cancers, perhaps reflecting the known increased dependency of RCC on iron. Intriguingly, RCC iron accumulation is reduced with pathological progression to metastasis despite increasing TfR1 iron importer overexpression and precisely opposite iron changes in adjacent renal epithelium, altogether suggesting a relative iron-deficient tumor state in patients with more advanced RCC. This reduction in tumor iron during progression is unique for any cancer studied to our knowledge and challenges the historical paradigm in which more iron is selectively advantageous in all phases of cancer evolution, supporting instead a novel model in which iron has distinct tissue-specific roles during RCC carcinogenesis and early tumorigenesis versus later progression. Future study is warranted to determine how these complex iron dynamics arise and interplay with the unique molecular genetics underlying RCC; and how they might translate into clinical therapeutics, including whether the relative iron deficiency in metastatic RCC tumors contributes to ferroptosis escape and/or increases tumor susceptibility to iron deprivation in a manner that is therapeutically exploitable.

## Data availability statement

The original contributions presented in the study are included in the article/**Supplementary Material**. Further inquiries can be directed to the corresponding author.

## Ethics statement

This study was reviewed and approved by Roswell Park Comprehensive Cancer Center Internal Review Board. Written informed consent for participation was not required for this study in accordance with the national legislation and the institutional requirements.

## Author contributions

Manuscript composition was performed by CG and EK, and editorial support was provided by KA, NS, BB, RD, JM, GS, KG, and BX. CG performed condition optimization for Prussian Blue staining. BX oversaw Prussian Blue scoring with assistance from CG. KA performed all statistical analyses. JM, GS, and KG provided intellectual support. EK was responsible for project conception and supervised all members of the research team and all experimental aspects. All authors contributed to the article and approved the submitted version.

## Funding

This research was supported by an American Cancer Society Institutional Research Grant and a Department of Defense Kidney Cancer Research Program Idea Award (W81XWH-20-1-0721). RPCCC patient tissue and Prussian Blue staining services were provided by the RPCCC Pathology Network Shared Resource, which is funded by the National Cancer Institute (NCI) Cancer Center Support Grant (CCSG) P30CA016056. Deidentified clinical data were provided by the RPCCC Biomedical Research Informatics Shared Resource, which is supported by the NCI CCSG, P30CA16056. Statistical support was provided by the RPCCC Biostatistics and Bioinformatics Shared Resource, which is supported by the NCI CCSG, P30CA016056.

## Conflict of interest

The authors declare that the research was conducted in the absence of any commercial or financial relationships that could be construed as a potential conflict of interest.

## Publisher’s note

All claims expressed in this article are solely those of the authors and do not necessarily represent those of their affiliated organizations, or those of the publisher, the editors and the reviewers. Any product that may be evaluated in this article, or claim that may be made by its manufacturer, is not guaranteed or endorsed by the publisher.

## Supplementary material

Supplementary Figure 1 Association of primary tumor iron levels with non-ccRCC patient survival outcomes. Iron levels in non-ccRCC patients were measured using Prussian Blue stain of the RPCCC RCC patient TMA set. Iron staining levels (H-score) in **(A)** primary tumor cells, **(B)** tumor microenvironment and **(C)** normal (non-neoplastic) kidney were tested for association with (left to right) metastasis-free survival, cancer-specific survival, and overall survival using Kaplan–Meier methodology.

## Abbreviations

IRP1/ACO1, iron regulatory protein 1; BMI, body mass index; ccRCC, clear cell RCC; chRCC, chromophobe RCC; DMT1/SLC11A2, divalent metal transporter 1; HIF-α, hypoxia-inducible factor-α; IRE, iron-responsive element; NCI, National Cancer Institute; non-ccRCC, non–clear cell renal cell carcinoma; PB, Prussian Blue; PTP, percentage of tissue positivity; pRCC, papillary RCC; RCC, renal cell carcinoma; RPCCC, Roswell Park Comprehensive Cancer Center; DMT1, divalent metal transporter 1; TfR1/TFRC/CD71, transferrin receptor 1; TMA(s), tissue microarray(s); VHL, von Hippel Lindau.
