# Supplementary figures and images for: Iron accumulation typifies renal cell carcinoma tumorigenesis but abates with pathological progression, sarcomatoid dedifferentiation, and metastasis
Source: Front Oncol. 2022 Aug 5;12:923043. doi: 10.3389/fonc.2022.923043 (PMC9389085; doi:10.3389/fonc.2022.923043)

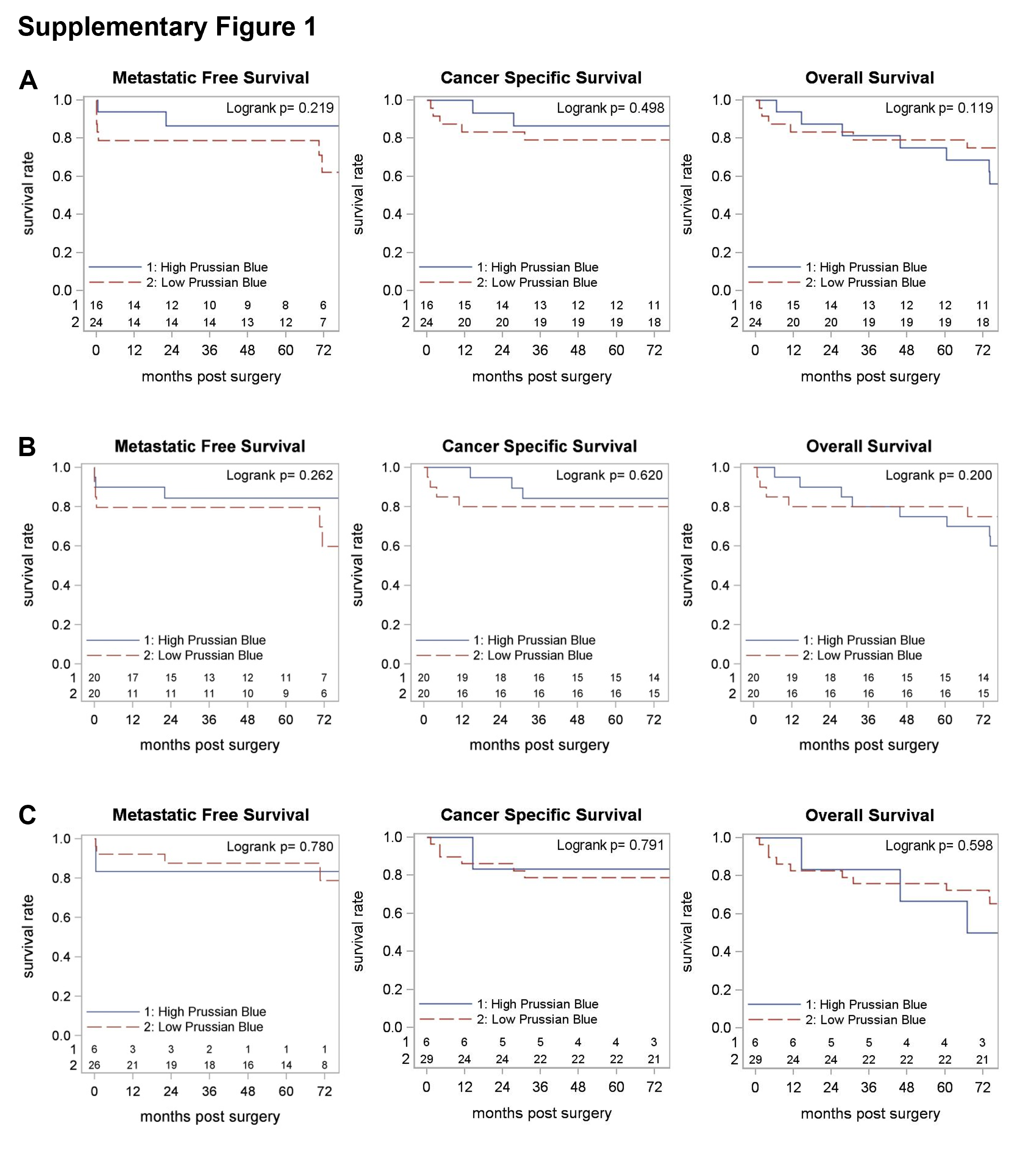

Supplement: Supplementary Figure 1 — Association of primary tumor iron levels with non-ccRCC patient survival outcomes. Iron levels in non-ccRCC patients were measured using Prussian Blue stain of the RPCCC RCC patient TMA set. Iron staining levels (H-score) in (A) primary tumor cells, (B) tumor microenvironment and (C) normal (non-neoplastic) kidney were tested for association with (left to right) metastasis-free survival, cancer-specific survival, and overall survival using Kaplan–Meier methodology. [file Image_1.tif]
