# Supplementary material for: Iron accumulation typifies renal cell carcinoma tumorigenesis but abates with pathological progression, sarcomatoid dedifferentiation, and metastasis
Source: Front Oncol. 2022 Aug 5;12:923043. doi: 10.3389/fonc.2022.923043 (PMC9389085; doi:10.3389/fonc.2022.923043)
Supplement: Supplementary file 2 [file Table_1.docx]

**Supplementary Table 1: Characteristics of patients in the RPCCC TMA set.**

|  | | **Patients,**  **n (%)** | **Tissues, n (%)** | | |
| --- | --- | --- | --- | --- | --- |
|  |  |  | **Primary**  **Tumor** | **Benign**  **Kidney** | **Metastasis** |
| Total number |  | 286 | 266 | 231 | 73 |
| Age, years | <= 60 | 154 (53.8) | 142 (53.4) | 118 (51.1) | 52 (71.2) |
|  | > 60 | 132 (46.2) | 124 (46.6) | 113 (48.9) | 21 (28.8) |
| Gender | Male | 178 (62.2) | 164 (61.7) | 146 (63.2) | 45 (61.6) |
|  | Female | 108 (37.8) | 102 (38.3) | 85 (36.8) | 28 (38.4) |
| Race | Caucasion | 267 (93.4) | 250 (94.0) | 217 (93.9) | 70 (95.9) |
|  | African American | 19 (6.6) | 16 (6.0) | 14 (6.1) | 3 (4.1) |
| Body Mass Index, kg/m^2^ | < 30 | 109 (50.0) | 118 (51.1) | 101 (51.0) | 15 (65.2) |
|  | >= 30 | 109 (50.0) | 113 (48.9) | 97 (49.0) | 8 (34.8) |
| Smoking History | Never | 122 (43.9) | 118 (45.4) | 101 (44.9) | 21 (29.6) |
|  | Any | 156 (56.1) | 142 (54.6) | 124 (55.1) | 50 (70.4) |
| Smoking Pack-Years | 0 | 122 (56.2) | 118 (52.9) | 101 (53.4) | 21 (60.0) |
|  | <= 30 | 55 (25.3) | 62 (27.8) | 50 (26.5) | 10 (28.6) |
|  | > 30 | 40 (18.4) | 43 (19.3) | 38 (20.1) | 4 (11.4) |
| Iron Supplementation | No | 122 (43.9) | 210 (94.2) | 180 (94.2) | 21 (87.5) |
|  | Yes | 156 (56.1) | 13 (5.8) | 11 (5.8) | 3 (12.5) |
| Anemia | No | 122 (56.2) | 92 (61.7) | 82 (62.6) | 2 (15.4) |
|  | Yes | 55 (25.3) | 57 (38.3) | 49 (37.4) | 11 (84.6) |
| Microcytic Anemia | No | 40 (18.4) | 115 (89.8) | 102 (90.3) | 3 (42.9) |
|  | Yes | 122 (43.9) | 13 (10.2) | 11 (9.7) | 4 (57.1) |
| Hemoglobin level, g/dL | < 13.3 | 63 (44.4) | 68 (45.6) | 59 (45.0) | 11 (84.6) |
|  | >= 13.3 | 79 (55.6) | 81 (54.4) | 72 (55.0) | 2 (15.4) |
| Hypertension | No | 64 (32.7) | 69 (32.9) | 61 (33.9) | 11 (47.8) |
|  | Yes | 132 (67.3) | 141 (67.1) | 119 (66.1) | 12 (52.2) |
| Histologic Subtype | ccRCC | 194 (67.8) | 200 (75.2) | 174 (75.3) | 33 (45.2) |
|  | pRCC | 26 (9.1) | 25 (9.4) | 23 (10.0) | 6 (8.2) |
|  | chRCC | 13 (4.5) | 12 (4.5) | 11 (4.8) | 0 (0) |
|  | Other RCC | 3 (1.0) | 3 (1.1) | 1 (0.4) | 1 (1.4) |
|  | Unspecified RCC | 39 (13.6) | 15 (5.6) | 12 (5.2) | 33 (45.2) |
|  | Oncocytoma | 11 (3.8) | 11 (4.1) | 10 (4.3) | 0 (0) |
| Tumor Grade | I | 14 (5.4) | 13 (5.2) | 12 (5.5) | 1 (1.6) |
|  | II | 126 (48.8) | 115 (46.2) | 104 (47.7) | 19 (31.1) |
|  | III | 72 (27.9) | 68 (27.3) | 57 (26.1) | 20 (32.8) |
|  | IV | 46 (17.8) | 53 (21.3) | 45 (20.6) | 21 (34.4) |
| Tumor Stage | pT1 | 130 (47.3) | 129 (50.6) | 113 (51.1) | 5 (6.8) |
|  | pT2 | 43 (15.6) | 44 (17.3) | 39 (17.6) | 8 (11.0) |
|  | pT3/4 | 70 (25.5) | 81 (31.8) | 68 (30.8) | 29 (39.7) |
|  | pTx | 32 (11.6) | 1 (0.4) | 1 (0.5) | 31 (42.5) |
| Tumor Size, cm | <= 4 | 96 (35.4) | 92 (35.4) | 84 (37.2) | 7 (11.7) |
|  | 4-7 | 84 (31.0) | 76 (29.2) | 63 (27.9) | 20 (33.3) |
|  | 7-10 | 49 (18.1) | 50 (19.2) | 46 (20.4) | 18 (30.0) |
|  | > 10 | 42 (15.5) | 42 (16.2) | 33 (14.6) | 15 (25.0) |
| Tumor Focality | Unifocal | 168 (88.0) | 176 (87.1) | 155 (88.1) | 16 (80.0) |
|  | Multifocal | 23 (12.0) | 26 (12.9) | 21 (11.9) | 4 (20.0) |
| Tumor Presence of Sarcomatoid | No | 217 (91.6) | 227 (90.1) | 196 (90.3) | 18 (69.2) |
|  | Yes | 20 (8.4) | 25 (9.9) | 21 (9.7) | 8 (30.8) |
| Tumor Sarcomatoid Percentage | 0 | 219 (93.2) | 229 (91.6) | 198 (92.1) | 20 (76.9) |
|  | <= 50 | 8 (3.4) | 10 (4.0) | 8 (3.7) | 2 (7.7) |
|  | > 50 | 8 (3.4) | 11 (4.4) | 9 (4.2) | 4 (15.4) |
