# Supplementary material for: Iron accumulation typifies renal cell carcinoma tumorigenesis but abates with pathological progression, sarcomatoid dedifferentiation, and metastasis
Source: Front Oncol. 2022 Aug 5;12:923043. doi: 10.3389/fonc.2022.923043 (PMC9389085; doi:10.3389/fonc.2022.923043)
Supplement: Supplementary file 3 [file Table_2.docx]

**Supplementary Table 2: Iron stain levels in metastatic primary tumors and metastases.**

| Patient histology | Iron stain incidence, n (%) | | | Iron stain level (mean H-score) | | |
| --- | --- | --- | --- | --- | --- | --- |
|  | Metastatic Primary  Tumor | Matched Metastasis | p-value | Metastatic Primary  Tumor | Matched Metastasis | p-value |
| All RCC | 21 (55.3%) | 14 (36.8%) | 0.17 | 8.6 | 4.1 | 0.12 |
| ccRCC | 17 (68.0%) | 12 (48.0%) | 0.27 | 12.8 | 6.1 | 0.19 |
| Non-ccRCC | 4 (30.8%) | 2 (15.4%) | 0.69 | 0.42 | 0.37 | 0.69 |
